# A new way to synthesize superconducting metal-intercalated C_60_ and FeSe

**DOI:** 10.1038/srep18931

**Published:** 2016-01-06

**Authors:** Yuuki Takahei, Keitaro Tomita, Yugo Itoh, Keishi Ashida, Ji-Hyun Lee, Naoki Nishimoto, Takumi Kimura, Kazutaka Kudo, Minoru Nohara, Yoshihiro Kubozono, Takashi Kambe

**Affiliations:** 1 Department of Physics, Okayama University, Okayama 700-8530, Japan; 2 Research Laboratory for Surface Science, Okayama University, Okayama 700-8530, Japan; 3 Research Centre of New Functional Materials for Energy Production, Storage and Transport, Okayama University, Okayama 700-8530, Japan; 4 Japan Science and Technology Agency, ACT-C, Kawaguchi 332-0012, Japan

## Abstract

Doping with the optimum concentration of carriers (electrons or holes) can modify the physical properties of materials. Therefore, improved ways to achieve carrier doping have been pursued extensively for more than 50 years. Metal-intercalation is one of the most important techniques for electron doping of organic / inorganic solids, and has produced superconductors from insulators and metallic solids. The most successful examples are metal-intercalated graphite and C_60_ superconductors. Metal intercalation has been performed using solid-reaction and liquid solvent techniques. However, precise control of the quantity of intercalants in the target solids can be difficult to achieve using these methods, as that quantity depends largely on the initial conditions. Here we report an electrochemical method for metal-intercalation, and demonstrate the preparation of superconductors using organic and inorganic materials (C_60_ and FeSe). The metal atoms are effectively intercalated into the spaces in C_60_ and FeSe solids by supplying an electric current between electrodes in a solvent that includes electrolytes. The recorded superconducting transition temperatures, *T*_c_’s, were the same as those of metal-intercalated C_60_ and FeSe prepared using solid-reaction or liquid solvent techniques. This technique may open a new avenue in the search for organic / inorganic superconductors.

Controlling the charge-carrier density in solids is one of the keys to controlling their physical properties. Several ways to control the charge-carrier density have been reported. The most common way is the partial substitution of a framework atom by a different atom with a different valence, which can then donate or accept electrons. Metal intercalation can donate electrons to solids, which shifts the Fermi level upward. Recently, the electric-double-layer transistor (EDLT) has attracted significant attention as a new way to control the carrier density at the interface of solids^1^,^2^,^3^,^4^,^5^, which can be achieved without any change of chemical composition or structure. Various novel physical properties such as superconductivity^1^,^2^,^3^, metal-insulator transition^4^ and ferromagnetism^5^ have been realized using the EDLT technique. In an EDLT, the carriers can accumulate around the interface, and can be controlled by an electric field. However, such simple processes as these have previously only been observed in EDLTs using inorganic materials, as described later.

Studies have also probed the carrier accumulation process in EDLTs, to achieve effective carrier accumulation. In particular, the process of carrier accumulation has been carefully investigated in graphene EDLTs^6^,^7^ because theorists predict that electron filling up to the van-Hove singularity (M-point) may produce novel physical properties such as chiral superconductivity^8^, CDW transition^9^ and ferromagnetism^10^. We recently used a capacitance measurement and electron spin resonance (ESR) to clarify the presence of some carrier accumulation processes in an EDLT that included an active layer of aromatic hydrocarbon molecule^11^,^12^. This EDLT displayed (1) electrostatic carrier accumulation at the interface between the hydrocarbon and the electric-double-layer, (2) electrochemical accumulation in the bulk of the hydrocarbon film and (3) the electrochemical reaction of hydrocarbon molecules. In the first and second processes, the carrier density can be reversibly changed with the bias voltage, while in the third process the carriers accumulated can be maintained even with the bias voltage switched off. Therefore, in the third process, the hydrocarbon molecule changed to a hydrocarbon radical cation. Thus, an EDLT that uses organic materials involves complex accumulation processes. From this experiment, we obtained a hint about how to accumulate carriers in solids and to modify their electronic structure. That is, the electrochemical reaction may be an effective way to induce novel physical properties.

This paper discusses how we applied the electrochemical approach to C_60_ and FeSe solids to accumulate electrons, *i.e.* to intercalate metal atoms. This leads to electron accumulation in the C_60_ molecules or FeSe layers. As a result, superconducting K_3_C_60_ and K- or Na-doped FeSe could be generated.

## Results

### Fabrication and characterisation of C_60_ superconductor

**Figure 1:**
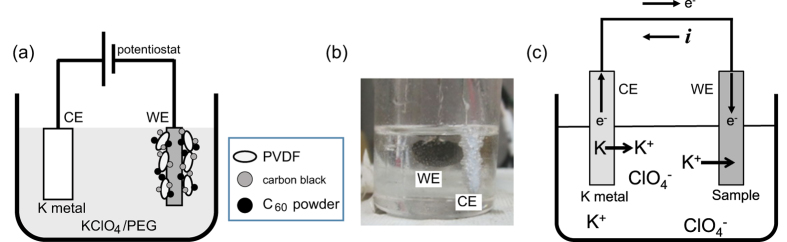
Schematic diagram of the electrochemical reaction cell. (**a**) Diagram and (**b**) photo of the electrochemical reaction cell used for the preparation of C_60_ superconductor. (**c**) Diagram for the electrochemical reaction process. This diagram corresponds to the potassium metal intercalation into C_60_ and FeSe.

Figure 1a,b show a diagram and photo of the electrochemical reaction cell. The working electrode (WE) consists of either K_x_C_60_ or C_60_, while the counter electrode (CE) is sodium (Na) or potassium (K) metal. We applied a constant voltage between the CE and WE during the electrochemical reaction. Firstly, we performed the electrochemical intercalation of K atoms in K_x_C_60_ that was synthesized by the usual solid-state reaction method^13^, because metallic/superconducting K_x_C_60_ should operate effectively as a good WE. Secondly, we tried to directly intercalate K atoms into semiconducting non-doped C_60_.

**Figure 2:**
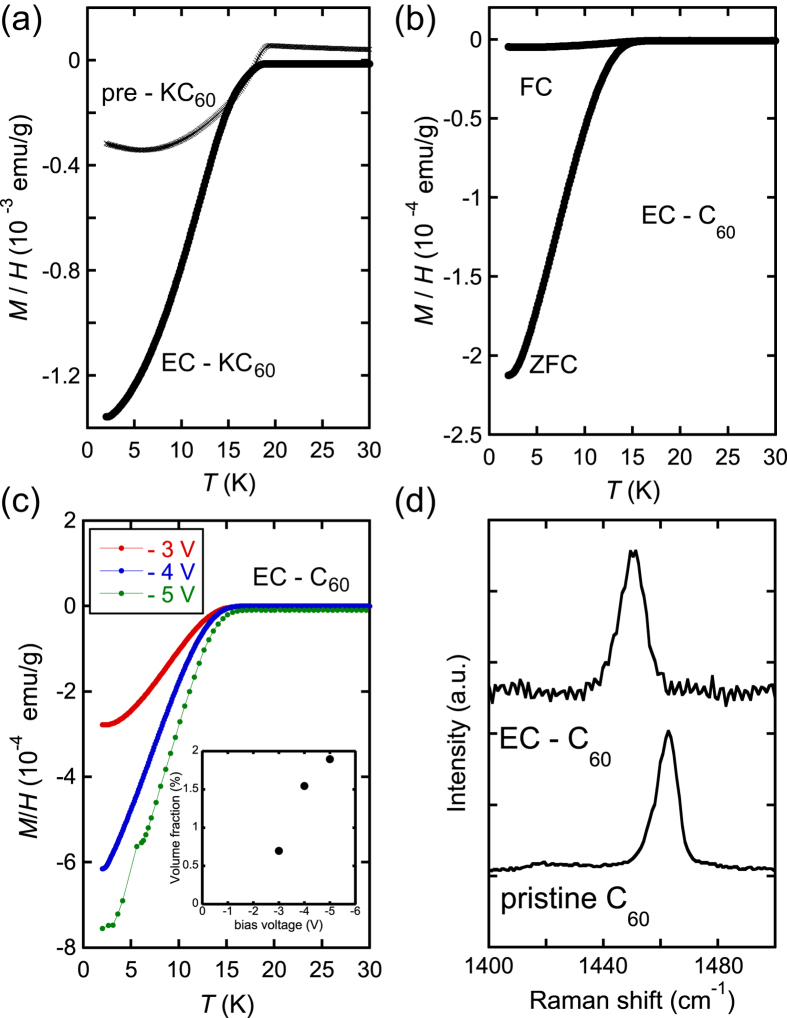
Superconductivity in electrochemically K-intercalated C_60_. (**a**) Temperature dependence of magnetic susceptibility for K_x_C_60_ precursor (pre-KC_60_), electrochemically intercalated K_x_C_60_ (EC−KC_60_) and (**b**) electrochemically intercalated C_60_ (EC−C_60_). (**c**) Bias voltage dependence of magnetic susceptibility for EC−C_60_ for a reaction time of 24 hours. The inset shows the bias voltage dependence of the superconducting volume fraction. In (**a–c**), the bias voltage was switched off during the *M*/*H* measurements. (**d**) Raman spectra for C_60_ and electrochemically intercalated EC−C_60_.

Figure 2a,b show the temperature dependence of magnetic susceptibility (*M*/*H* – *T, M: magnetization, H: applied magnetic field, T: temperature*) for three samples (pre-KC_60_, EC-KC_60_ and EC-C_60_); “pre-KC_60_” refers to K-intercalated C_60_ prepared by the solid-state reaction, “EC-KC_60_” refers to the sample synthesized electrochemically from pre-KC_60_, and “EC-C_60_” refers to the sample synthesized electrochemically from C_60_. For electrochemical intercalation of the pre-KC_60_ pellet sample, a voltage of −5 V was applied to the CE for 4 h. As seen from Fig. 2a, the superconducting volume faction was clearly increased from 1% (pre-KC_60_) to 3% (EC-KC_60_) through the electrochemical intercalation of K atoms. The temperature dependence of magnetic susceptibility measured under the zero-field-cooled (ZFC) protocol for EC-KC_60_ shows a clear drop at ~18 K, showing that the onset superconducting transition temperature, *T*_C_, is consistent with that, 19.3 K, of the K_3_C_60_ superconductor^14^ and that, 18.5K, of pre-KC_60_.

Figure 2b shows the *M*/*H* – *T* plot under field-cooled (FC) and ZFC protocols for the EC-C_60_ sample. In this case, we used the pellet of PVDF/C_60_/CB mixture for the WE because the conductivity must be increased, where the C_60_ powder and carbon black powder (CB) were mixed with polyvinylidene difluoride (PVDF) as a binder. We applied a voltage of −3 V for 8 h. The *T*_C_ in the EC-C_60_ sample was found to be ~17.5 K.

To see the bias voltage dependence of the superconductivity, we applied different bias voltages between the CE and WE. Figure 2c shows the bias voltage dependence of magnetic susceptibility for the semiconducting C_60_ sample (EC-C_60_). In this experiment, we used reaction times of 24 h. After applying a bias voltage of −3 V for 24 h, the temperature dependence of magnetic susceptibility was measured (see red solid circle (−3 V)), and then a bias voltage of −4 V was applied for 24 h and the magnetic susceptibility was measured again (blue solid circle). The experimental procedure was repeated up to −5 V (green solid circle). During the measurement of temperature dependence of magnetic susceptibility, no bias voltage was applied. The superconducting volume fraction increases with increasing bias voltage, as seen from Fig. 2c. As seen from the inset of Fig. 2c, the superconducting volume fraction increased linearly with increasing bias voltage. The number of K ions dissolved in the electrolytic solution would be expected to increase with an increasing bias voltage, leading to the observed increase in the metal-intercalated (or superconducting) domain in the WE. Thus, an electrochemical reaction has intercalated K atoms into C_60_ to produce the superconducting phase.

To investigate carrier donation to a C_60_ molecule, a Raman scattering experiment was carried out at room temperature. It is well established that the electron-filling of the *t*_1u_-orbital of C_60_ can be clarified by the negative shift of the centro-symmetric Ag(2) mode of the C_60_ molecule (1469 cm^−1^ for pure C_60_)^15^. Figure 2d shows Raman spectra of both pristine C_60_ and the EC-C_60_ sample. Ag(2) peak was recorded for the EC-C_60_ at 1450 cm^−1^, where EC-C_60_ refers to the sample after the bias-voltage application of −5 V (Fig. 2c). This result implies that the valence of EC-C_60_ is 3.1, because a negative shift by 6 cm^−1^ per 1 electron-donation on C_60_ should be observed. The value of 3.1 is consistent with the valence of the C_60_ molecule in superconducting metal-doped C_60_ (M_3_C_60_; M = alkali metal atom). As the Raman scattering experiment probes the electronic state around the surface, the doped main phase there would be M_3_C_60_. This experiment suggests that no other phase exists with a metal concentration greater than 3, *i.e.*, M_4_C_60_ or M_6_C_60_. Thus, superconducting K_3_C_60_ was prepared by this electrochemical reaction. This is the first preparation of superconducting metal-doped C_60_ using any technique other than solid-state or liquid NH_3_ chemical reactions

**Figure 3:**
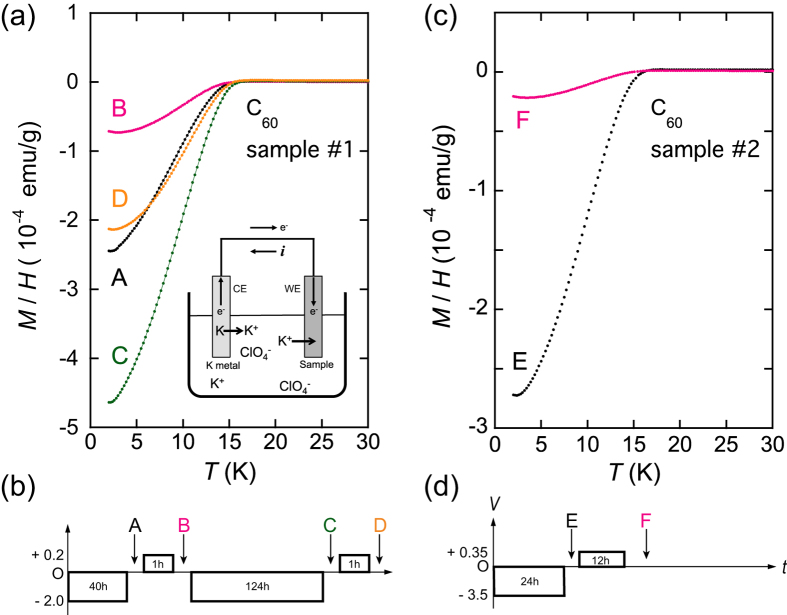
Reversibility of superconductivity in C_60_ by the application of bias voltage with different polarity. (**a,c**) Temperature dependence of magnetic susceptibility for EC−C_60_ for the application of bias voltage with different polarity. Two pellet samples of C_60_ were used (sample #1 and #2). (**b**) Time-sequence of bias voltage in sample #1. Firstly, a bias voltage of −2.0 V was applied for 40 h, and, at A, the temperature dependence of magnetic susceptibility was measured (see black solid circle in (**a**)). Then, a bias voltage of +0.2 V was applied for 1 h and the magnetic susceptibility was measured again (B). (**d**) Time-sequence of bias voltage in sample #2.

One of distinct advantages of the electrochemical technique is to charge or discharge carriers by applying the reversible bias voltage. We demonstrates reversibility of superconductivity in C_60_ by the application of bias voltage with different polarity (see Fig. 3). After the appearance of superconductivity due to the negative bias voltage, the application of positive bias voltage leads to a reduction or disappearance of superconducting fraction. This can repeat itself completely. As the superconductivity should be founded on the K-metal intercalation into the parent C_60_, the reduction of superconductivity implies a de-intercalation of K-metal. Accordingly, the intercalation / de-intercalation of K-metal atoms can be reversibly controlled by the bias voltage.

### Fabrication and characterisation of FeSe superconductors

We applied electrochemical intercalation to the layered inorganic material, FeSe, the *T*_C_ of which is 8 K^16^. It is well known that alkali and alkali-earth metal intercalation in the FeSe solids can lead to higher *T*_C_’s than that, 8 K, of pristine FeSe. A maximum *T*_C_ of 46 K is currently obtained in ammoniated Na-doped FeSe^17^. Here, in addition to the metal atoms, NH_3_ is incorporated between the FeSe layers. It is proposed that the *T*_C_ is closely correlated with the FeSe interlayer distance, *d*^18^, which implies that an increase in two-dimensionality (2D) produces a higher *T*_C_. Recent theoretical study of HfNCl suggests that the improvement of Fermi-surface-nesting increases spin-fluctuation to strengthen pair coupling^19^. This scenario may be applied to FeSe and similar van der Waals materials. Thus, the intercalation of metal atoms into FeSe layers is very attractive for the realization of high-*T*_C_ superconductors. Furthermore, the parent material, FeSe, has a simple structure consisting solely of conducting layers that are coupled through van der Waals interaction, *i.e.*, a very flexible structure. Therefore, the effective intercalation of metal atoms into the FeSe system by the electrochemical method was judged to be achievable.

**Figure 4:**
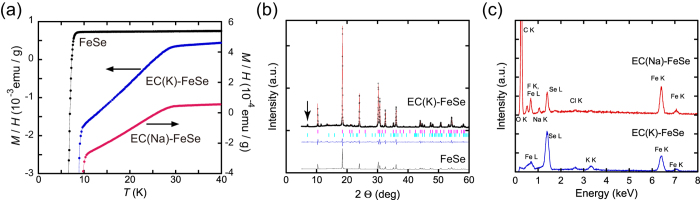
Superconductivity in electrochemically K- and Na-intercalated FeSe. (**a**) Temperature dependence of magnetic susceptibility for parent FeSe and electrochemically intercalated FeSe (EC(K)-FeSe and EC(Na)- FeSe). (**b**) XRD spectra for parent FeSe and EC(K)-FeSe at room temperature. For the EC(K)-FeSe, the black cross indicates the experiments and the red line indicates the fitting result based on the Le Bail analysis. Below the XRD spectrum, the blue and pink vertical bars indicate expected diffraction positions for the parent FeSe and the ThCr_2_Si_2_-type structure. The (002) peak for the ThCr_2_Si_2_-type structure was indicated by the arrow. (**c**) EDX spectra for EC(K)-FeSe and EC(Na)-FeSe. In the case of EC(Na)-FeSe, the peaks of C, O and F are observed because of the use of PVDF as the binder.

In the experiment for K-intercalation, the FeSe pellet sample was used as the WE, and K metals were used as the CE. In the case for Na-intercalation, we used the PVDF/FeSe mixture spread on an Al-foil as the WE. A bias voltage of −3 V was applied for 24 hours. Figure 4a shows the *M*/*H* – *T* plot in the ZFC protocol for a pristine FeSe sample (black dot), electrochemically K-intercalated FeSe (blue dot) and electrochemically Na-intercalated FeSe (red dot), respectively, which are denoted “FeSe”, “EC(K)-FeSe” and “EC(Na)-FeSe”, respectively. For the EC(K)-FeSe, clear drops in the *M*/*H*—*T* plot are found at 9 K and 29 K. The 9 K superconducting transition is due to the residual non-intercalated FeSe phase, while the 29 K superconducting transition is consistent with that, *T*_C_ = 30 K^20^, of K_x_FeSe prepared by the solid-state reaction and that, *T*_C_ = 31 K^18^,^21^, of (NH_3_)_y_K_x_FeSe prepared by the liquid NH_3_ technique, respectively. These results support the intercalation of K atoms into FeSe layers. Here, we must ask whether the electrolyte is also incorporated in the space between FeSe layers together with K atoms. This is fully discussed in the subsequent section.

The superconducting volume fraction for EC(K)-FeSe is estimated to be 11% at 10 K. Accordingly, we have succeeded in synthesizing a K-doped FeSe phase using electrochemical intercalation. The magnetic susceptibility of the EC(Na)-FeSe sample is also shown in Fig. 4a. The superconducting volume fraction for EC(Na)-FeSe is estimated to be 2% at 10 K. The temperature dependence of magnetic susceptibility of EC(Na)-FeSe shows a *T*_C_ as high as 30 K, which is lower than that, *T*_C_ = 46 K^17^, previously reported for Na_x_Fe_2_Se_2_, and is close to that, *T*_C_ = 32 K^22^, of the low-*T*_C_ phase of (NH_3_)_y_Na_x_FeSe found recently.

### x-ray diffraction and composition of electrochemically K-intercalated FeSe superconductor

To confirm the intercalation of metal into solid FeSe, we performed x-ray diffraction measurements with synchrotron radiation (KEK-PF, BL-8B). Figure 3b shows the x-ray diffraction pattern for EC(K)-FeSe powder measured with λ=1.0004(8) Å. As shown in Fig. 4b, the (002) peak characteristic of the ThCr_2_Si_2_-type structure (body-centred tetragonal: space group No.139) was clearly observed in EC(K)-FeSe; the (002) peak is not observed in pure FeSe (see Fig. 3b). The existence of the (002) peak is attributed to the expansion along the *c*-axis due to K-intercalation between the FeSe layers. The lattice parameters for EC(K)-FeSe were determined, based on the the Le Bail fitting analysis (see in Fig. 4b). The *a* and *c* were determined to be 3.71 Å and 16.4 Å, respectively. The estimated *c* is much larger than that, *c* = 14.0367(7) Å, of K_x_Fe_2_Se_2_^19^ while it is close to that, *c* = 16.16(5) Å, of (NH_3_)_y_KFe_2-δ_Se_2_^21^. These results suggested that not only K atoms but also electrolyte may have been incorporated in the FeSe solid.

To confirm the exact intercalation of both K atoms and electrolyte molecules, we used energy dispersive x-ray spectroscopy (EDX) on the EC(K)-FeSe and EC(Na)-FeSe samples (see Fig. 4c). From the EDX spectra shown in Fig. 4c, the composition of the EC(K)-FeSe was estimated to be K_0.19_Fe_0.96_Se_1_. Here, only the peaks of Fe, Se and K are observed, suggesting no incorporation of electrolyte such as ClO_4_. Therefore, in spite of the expansion of *c* (the FeSe interlayer distance), the electrolyte molecule was not incorporated in the EC(K)-FeSe. It is of interest that electrochemical K-intercalation increases the FeSe layer separation without incorporating any electrolyte molecules (or any solvent). In the case of EC(Na)-FeSe, the peaks of Fe, Se and Na are observed, suggesting the Na-intercalation into FeSe.

## Discussion

We have demonstrated the electrochemical synthesis of superconducting phases of C_60_ and FeSe by intercalating alkali metal atoms into solid C_60_ and FeSe. Thus, the electrochemical approach is an effective way to produce superconducting phases.

The superconducting volume fraction of the electrochemically synthesized C_60_ and FeSe tends toward saturation as the reaction time increases. Raman scattering from the C_60_ indicates that the superconducting K_3_C_60_ phase is formed at the surface. The formation of the stable superconducting phase at the surface may prevent the metal atoms from further intercalating into the sample, thus causing the observed saturation of the superconducting volume fraction. This is likely to be an important consideration to realize higher superconducting volume fraction when attempting to achieve uniform penetration of the metal atoms into the bulk region.

Electrochemically Na-intercalated FeSe (EC(Na)-FeSe) shows a *T*_C_ as high as 30 K, which is close to that of the low-*T*_C_ phase of (NH_3_)_y_Na_x_FeSe (*T*_C_ = 32 K^22^). (NH_3_)_y_Na_x_FeSe has multiple superconducting phases depending on the concentrations of Na and NH_3_. Guo *et al.* reported three superconducting phases of (NH_3_)_y_Na_x_FeSe: an NH_3_-poor phase (*T*_C_ = 45 K), an NH_3_-rich phase (*T*_C_ = 42 K), and an NH_3_-free phase (*T*_C_ = 37 K)^23^. Very recently, Zheng *et al.* reported the presence of an NH_3_-poor low-*T*_C_ phase (*T*_C_ = 32 K), which was formed in a low concentration of Na^24^. This electrochemical synthesis produced the 30 K superconducting phase, whose *T*_C_ is close to that of the NH_3_-poor low-*T*_C_ phase (*T*_C_ = 32 K)^24^. Therefore, several *T*_C_ phases besides the low-*T*_C_ phase can be produced by adjusting the concentration of Na by varying the reaction time and/or initial concentration of Na during the electrochemical process. The precise control of the dopant concentration in M_x_FeSe will be a future issue.

The metal intercalation in the electrochemical process appears to be similar to the redox reaction in Li-ion batteries. Raman scattering of the electrochemically prepared superconducting C_60_ proved the donation of electrons to the *t*_1u_-levels of C_60_. As seen from Fig. 1c, the reduction of C_60_ and FeSe (or formation of the anions) during the electrochemical process involves the intercalation of K^+^ as the counter ion, since K^+^ is the sole cation in the electrolyte. In fact, K atoms are detected in the EDX spectra for the electrochemically prepared FeSe (EC(K)-FeSe) (see Fig. 4c).

The efficiency of normal metal-intercalation procedures such as high-temperature annealing and liquid solvent methods should be correlated with the ionization potential of metal atoms and the electron affinity of the target materials. Lower ionization potential of the dopant and higher electron affinity of the host material could be expected to produce effective metal intercalation in these reaction processes. On the other hand, in the electrochemical reaction, applying a positive voltage to the CE (K metal) forces the removal of electrons from K metal, as shown in Fig. 1c, *i.e.*, K metal in the CE is oxidized to supply K^+^ to the electrolyte solution. Electrons are supplied to C_60_ solid (WE) through the electric circuit (see Fig. 1c). The K^+^ migrates from the CE to the WE in the electrolyte solution so that K^+^ is intercalated into the C_60_ and FeSe solids. Therefore, the concentration of K^+^ is maintained at a constant level in the electrolyte solution until the K metal is exhausted. As a result, the electrochemical reactions of C_60_ and FeSe can be expressed as ‘
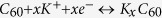
’ and ‘
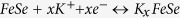
’, respectively.

Our experiments suggest the practical utility of the electrochemical reaction for the intercalation of metal atoms into C_60_ and FeSe. The electrochemical technique has the advantage of controlling the metal-concentration by the bias voltage. The reversible bias voltage yields the intercalation / de-intercalation of metal atoms, leading to appearance / disappearance of superconductivity. Intercalation of alkali-earth atom should be more effective because they can donate twice as many electrons as alkali metal atoms. In fact, Mg intercalation into a layered nitride (ZrNCl) using the electrochemical reaction produced a new superconducting phase^25^. Therefore, this technique may enable the control of the Fermi level by donating electrons to target materials. Layered materials with large interstitial sites are promising candidates for producing novel superconductors using the electrochemical process.

## Methods

### Electrochemical intercalation

Figure 1 shows a diagram and photo of the electrochemical cell used in this experiment. The electrochemical reaction for metal intercalation was performed using three electrodes; CE, reference electrode (RE) and WE. The CE is formed with alkali metal, which is an intercalant. The RE refers to the standard voltage of a potentiostat as shown below, and is composed of the same metal as the CE. The WE consists of the target material for the metal intercalation, C_60_ or FeSe, which is connected by a Pt wire. The electrolyte for K intercalation was made by dissolving KClO_4_ in polyethylene glycol (PEG) (KClO_4_: PEG = 1: 20), while the electrolyte for Na intercalation was made by dissolving NaCl in PEG (NaCl: PEG = 1: 20). A potentiostat (HA151 and/or HAL3001, Hokuto Denko Co. Ltd.) was used to apply a constant voltage between CE and WE. The voltage was varied from 0 to –5 V during the electrochemical reaction. The electrochemical cell (see Fig. 1) was placed in an incubator set at 30 °C. The detail of the electrochemical reaction is described in the Results section. After the electrochemical reaction, the sample prepared was introduced into a measurement cell in an Ar-filled glove box (O_2_, H_2_O < 0.1 ppm) for the measurement of the *M*/*H*.

### Measurements of physical properties

The *M*/*H* values of the sample were measured by a squid magnetometer (MPMS2 or MPSM3, Quantum Design Co. Ltd.). The *T*_C_ value was estimated from the onset temperature. To determine the valence of C_60_, Raman scattering experiments were performed using a Raman spectrometer (NRS-5000, JASCO Co. Ltd). We recorded the synchrotron radiation x-ray powder diffraction pattern at the BL-8B in KEK-PF, Japan; the wavelength of the x-ray beam was 1.0004(8) Å. The quantitative composition of the K-doped FeSe sample was analysed by electron dispersive x-ray (EDX) spectrometer equipped with a scanning microscope (SEM-EDAX Genesis XM_2_) (VE-9800SP, Keyence Co. Ltd).

## Additional Information

**How to cite this article**: Takahei, Y. *et al.* A new way to synthesize superconducting metal-intercalated C_60_ and FeSe. *Sci. Rep.* **6**, 18931; doi: 10.1038/srep18931 (2016).
